# Class-level aggregation obscures clinically relevant heterogeneity in anti-amyloid antibody trials: comments on a Cochrane review by individual members of the EADC

**DOI:** 10.1016/j.tjpad.2026.100598

**Published:** 2026-05-18

**Authors:** Kristian Steen Frederiksen, Mercè Boada, Lutz Frölich, Milica Kramberger, Nikolaos Scarmeas, Everard Vijverberg, Pieter Jelle Visser, Gunhild Waldemar, Eric Westman, Henrik Zetterberg, Sebastiaan Engelborghs, Frank Jessen

**Affiliations:** a Danish Dementia Research Centre, Department of Neurology, Copenhagen University Hospital - Rigshospitalet, Copenhagen, Denmark; b Department of Clinical Medicine, Faculty of Health and Medical Sciences, University of Copenhagen, Copenhagen, Denmark; c Ace Alzheimer Center Barcelona – Universitat Internacional de Catalunya, Barcelona, Spain; d Networking Research Center on Neurodegenerative Diseases (CIBERNED), Instituto de Salud Carlos III, Madrid, Spain; e Department of Geriatric Psychiatry, Central Institute of Mental Health, Medical Faculty Mannheim, Heidelberg University, Mannheim, Germany; f Department of Neurology, University Medical Center, Faculty of Medicine, University of Ljubljana, Ljubljana, Slovenia; g Division of Clinical Geriatrics, Department of Neurobiology, Care Sciences and Society, Karolinska Institutet, Stockholm, Sweden; h 1st Department of Neurology, Aiginition Hospital, National and Kapodistrian University of Athens Medical School GR, Athens, Greece; i Department of Neurology, Taub Institute for Research in Alzheimer's Disease and the Aging Brain, The Gertrude H. Sergievsky Center, Columbia University, New York, NY, USA; j Alzheimer Center Amsterdam, Neurology, Vrije Universiteit Amsterdam, Amsterdam UMC location VUmc, Amsterdam, Netherlands; k University Hospital of Maastricht, Department of Neuropsychiatry and Old Age Psychiatry, 6229 HX Maastricht, The Netherlands; l Theme Inflammation and Ageing, Karolinska University Hospital, Stockholm, Sweden; m Division of Clinical Geriatrics, Department of Neurobiology, Care Sciences, and Society, Karolinska Institutet, Stockholm, Sweden; n Department of Psychiatry and Neurochemistry, Institute of Neuroscience and Physiology, University of Gothenburg, Gothenburg, Sweden; o Clinical Neurochemistry Laboratory, Sahlgrenska University Hospital, Mölndal, Sweden; p UK Dementia Research Institute at UCL, London, UK; q Department of Neurodegenerative Disease, UCL Queen Square Institute of Neurology, London, UK; r Department of Pathology and Laboratory Medicine, University of Wisconsin School of Medicine and Public Health, Madison, WI, USA; s Wisconsin Alzheimer's Disease Research Center, University of Wisconsin School of Medicine and Public Health, Madison, WI, USA; t Centre for Brain Research, Indian Institute of Science, Bangalore, India; u Department of Neurology and Bru-BRAIN, Universitair Ziekenhuis Brussel and NEUR Research Group, Center for Neurosciences (C4N), Vrije Universiteit Brussel (VUB), Brussels, Belgium; v Department of Psychiatry, Medical Faculty, University of Cologne, Cologne, Germany; w German Center for Neurodegenerative Diseases (DZNE), Bonn, Germany; x Excellence Cluster on Cellular Stress Responses in Aging-Associated Diseases (CECAD), University of Cologne, Cologne, Germany

**Keywords:** Anti-amyloid, Alzheimer's disease, Monocloncal antibodies

The recent Cochrane metanalysis of anti-amyloid therapies for Alzheimer’s disease represents an attempt to synthesize an evolving evidence base [1] The rigor of the Cochrane methodology, including systematic study identification, risk-of-bias assessment, and transparent statistical approaches, may provide a valuable foundation for evaluating therapeutics. However, the central issue is not methodological formality but whether the analytical framework is appropriate for a therapeutic field that has evolved substantially over time.

The central misconception of the review relates to the decision to include all amyloid-beta targeting monoclonal antibodies in one metanalysis. While such aggregation increases statistical power, it obscures the substantial drug-to-drug heterogeneity, and differences regarding trial designs and developmental eras. The trajectory from early agents such as bapineuzumab, solanezumab, and crenezumab to more recent antibodies like lecanemab and donanemab, reflect not only incremental optimization but also fundamental evidence-based shifts in target engagement, dosing strategies, patient selection, and biomarker integration [2]. These differences are highly relevant to clinical outcomes.

Early-generation antibodies were developed in a context where amyloid biology was less understood, amyloid positivity was not consistently confirmed before enrollment [3], and dosing was limited by concerns regarding amyloid-related imaging abnormalities [4]. These constraints contributed to modest or absent clinical effects, reflected in negative trial outcomes, which are now widely interpreted as reflecting suboptimal target engagement rather than definitive evidence against the therapeutic hypothesis. In contrast, later-generation antibodies, specifically lecanemab [5] and donanemab [6], were designed to preferentially bind aggregated forms of amyloid, deployed at higher doses, and tested in biomarker-confirmed populations at earlier disease stages. These advances are not merely incremental; they represent a qualitative shift in how the amyloid hypothesis has been operationalized in clinical trials.

By pooling results across this spectrum, the review implicitly assumes a degree of class homogeneity that does not align with the underlying biology or trial methodology. The review's subgroup analyses show that heterogeneity is not hypothetical. If treatment effects vary by antibody type, then a class-average estimate is not a neutral summary but a potentially misleading one. This approach dilutes signals from more recent agents with negative or inconclusive findings from earlier compounds. The review considers together 9 different drugs from 17 different studies, from which only 2 drugs were approved. Therefore, the aggregated estimates will underestimate the efficacy of currently approved therapies while overemphasizing the failures of prior generations. A stratified analysis, reflecting developmental stage or key pharmacodynamic characteristics (such as plaque clearance capacity), is needed to provide an informative synthesis.

This concern also affects external validity. Only a minority of the included studies correspond to therapies that are now relevant to practice (lecanemab and donanemab). Broad conclusions about “anti-amyloid therapies” derived predominantly from compounds that never entered clinical use should be interpreted cautiously when applied to currently available treatments. Aggregating across approved and non-approved therapies without differentiation conflate fundamentally different evidentiary standards. It also leads to conclusions about efficacy and safety that are disproportionately influenced by agents that failed to meet regulatory benchmarks. Therefore, the conclusions that removal of amyloid is not associated with clinically meaningful results and that “The findings of the review could therefore be an important support for decision‐making in clinical practice” [1] cannot be made and are even clearly misleading.

A more informative approach would have been to present separate analyses for approved versus non-approved therapies. Such stratification allows readers to better interpret how the evidence base relates to current clinical decision-making. Without this distinction, there is a risk that the conclusions are perceived as applying uniformly across all agents, despite substantial differences in their evidentiary support.

Another major concern relates to the definition and application of clinical relevance. The review adopts thresholds for clinical meaningfulness that are derived from a limited number of anchoring studies defining the Minimal clinically important difference (MCID) [7]. Anchoring studies are inherently context-dependent, influenced by patient populations, disease stages, outcome measures, and methodological assumptions. These concerns are also reflected in a recent commentary in Lancet Regional Health [8] by authors from the European Medicines Agency involved in the evaluation of lecanemab and donanemab: “A decision on clinical relevance based solely on the MCID therefore did not appear adequate in this case, hence other lines of evidence were considered.”

The strength of the Cochrane approach lies in its methodology, which facilitates reproducibility and comparability. However, when applied to a therapeutic area undergoing rapid scientific evolution and ignoring scientific insights, strict aggregation can inadvertently obscure clinically meaningful differences between interventions, and result in erroneous conclusions that could cause patient harm. As it stands, our view is that Cochrane should reconsider their report for withdrawal.

## Funding

None.

MB reports no disclosures

LF received research grants from Hoffmann-LaRoche, Hector II Foundation, Dietmar Hopp Foundation, EU-H2020–2017 (Grant no. 779237); EU-IMI-2019 (grant no. 806999); EU-H2022 (Grant No. 101120706), EU-H2024 (Grant no. 101156500), he serves on an advisory boards, participated in educational and speaker activities for Avanex, Biogen, BioVie, Bristol-Myers Squibb, Charles-River Ass., Eli Lilly, Eisai, GE Healthcare, Grifols, Janssen-Cilag, Janssen Research, Neurimmune, Noselab, NovoNordisk, Roche, TauRX, Schwabe, DerCampus, Medscape, Medfora, FOMF and has served on data monitoring committees for Neuroscios, ReMynd, Otsuka/Avanir, Vivoryon.

MK participated in educational and speaker activities for Biogen, Eli Lilly, Roche, Abbvie and Stada.

NS reports no disclosures.

EV reports having received consultancy fees (paid to the university) from New Amsterdam Pharma, Treeway, ReMynd, Vivoryon, Biogen, Vigil Neuroscience, ImmunoBrain Checkpoint, Muna Therapeutics, Eisai, Eli Lilly, CogRx, Therini, UCB, and Roche.

Within his university affiliation, he is the principal investigator of studies for DIAN, AC Immune, Alnylam, CogRx Therapeutics, New Amsterdam Pharma, Janssen, UCB, Roche, Vivoryon, ImmunoBrain, GSK, MSD, Biogen, Alector, Eli Lilly, AriBio, Fuji Film Toyama, and GemVax.

PJV reports no disclosures.

GW reports no disclosures.

EW reports no disclosures.

HZ has served at scientific advisory boards and/or as a consultant for Abbvie, Acumen, Alamar, Alector, Alzinova, ALZpath, Amylyx, Annexon, Apellis, Artery Therapeutics, AZTherapies, Cognito Therapeutics, CogRx, Denali, Eisai, Enigma, LabCorp, Merck Sharp & Dohme, Merry Life, Nervgen, New Amsterdam, Novo Nordisk, Optoceutics, Passage Bio, Pinteon Therapeutics, Prothena, Quanterix, Red Abbey Labs, reMYND, Roche, Samumed, ScandiBio Therapeutics AB, Siemens Healthineers, Triplet Therapeutics, and Wave, has given lectures sponsored by Alzecure, BioArctic, Biogen, Cellectricon, Fujirebio, LabCorp, Lilly, Novo Nordisk, Oy Medix Biochemica AB, Roche, and WebMD, is a co-founder of Brain Biomarker Solutions in Gothenburg AB (BBS), which is a part of the GU Ventures Incubator Program, and is a shareholder of CERimmune Therapeutics (outside submitted work).

SE received research grants from EU IHI, EU IMI, GSKE/FMRE, Innoviris, Interreg Vlaanderen-Nederland, Research Foundation Flanders (FWO), VLAIO, (all paid to institution), consulting fees from Biogen, Eli Lilly, Eisai, Icometrix, Novartis, Remynd (all paid to institution), personal consulting fees from Biogen and Roche, travel support to institution from Biogen, Patent EP3452830B1 (institution). SE serves / served as member of SMB/SAB for EU-H2020 project RECAGE, and as chair of the DSMB of PRImus-AD (all paid to institution). VP of Belgian Dementia Council and co-chair of EADC (unpaid).

FJ serves on advisory boards, participated in educational and speaker activities for Abbvie, Eisai, Eli Lilly, GE Healthcare, Grifols, Janssen Cilag, Novonordisk, Roche, and serves on a data monitoring committee for AC Immune.

## Ethical statement

Not applicable.

## Data availability

Not applicable.

## CRediT authorship contribution statement

**Kristian Steen Frederiksen:** Conceptualization, Writing – original draft, Writing – review & editing. **Mercè Boada:** Writing – original draft, Writing – review & editing. **Lutz Frölich:** Writing – original draft, Writing – review & editing. **Milica Kramberger:** Writing – original draft, Writing – review & editing. **Nikolaos Scarmeas:** Writing – original draft, Writing – review & editing. **Everard Vijverberg:** Writing – original draft, Writing – review & editing. **Pieter Jelle Visser:** Writing – original draft, Writing – review & editing. **Gunhild Waldemar:** Writing – original draft, Writing – review & editing. **Eric Westman:** Writing – original draft, Writing – review & editing. **Henrik Zetterberg:** Writing – original draft, Writing – review & editing. **Sebastiaan Engelborghs:** Conceptualization, Writing – original draft, Writing – review & editing. **Frank Jessen:** Conceptualization, Writing – original draft, Writing – review & editing.

## Declaration of competing interest

The authors declare that they have no known competing financial interests or personal relationships that could have appeared to influence the work reported in this paper.

KSF is or has been member of advisory boards or acts or has acted as consultant for Eisai, Novo Nordisk, Roche Diagnostics, Eli Lilly, AbbVie, BioArctic, Roche (remuneration paid to institution), Imprint Medicine (no renumeration), has speaking engagement for Eisai, BioArctic, Eli Lilly, Novo Nordisk, Roche Diagnostics (remuneration paid to institution), Best Practice Nordic (personal renumeration) is or has been principal investigator in clinical trials for Biogen, Novo Nordisk, Roche, Roche Diagnostics (remuneration paid to institution), has produced educational material for Medscape (personal remuneration), has provided consultation for Guidepoint and Neurocode (personal remuneration), is Editor-in-Chief for Alzheimer’s Research and Therapy (Springer - Nature) (personal remuneration) and has received research funding from Aase og Ejner Danielsens Fond, Alzheimer Forskningsfonden, A.P. Møller fonden, Beckett fonden, C2N, DANMODIS, Ellen Mørch Fonden, ERA-PERMED, Fonden for Neurologisk Forskning, Grosserer F.L.Foghts Fond, Harboefonden, Hertzfonden, IHI, Innovationsfon-den, Jascha Fonden, KID fonden, Kong Christian den Tiendes Minde-fond, Overretssagfører L. Zeuthens Mindefond, Parkinsonforeningen, Rigshospitalets Forskningspulje.
